# A monocyte-derived blood transcriptomic signature reveals systemic immunosuppression in HCC and partial reversal following curative therapy

**DOI:** 10.3389/fimmu.2025.1717978

**Published:** 2026-01-21

**Authors:** Liang Zhou, Ahmed Alaswad, Aditi Kumthekar, Dominik Machtens, Yuesi Xi, Bibiana Costa, Cheng-Jian Xu, Thomas Wirth, Yang Li

**Affiliations:** 1 Centre for Individualised Infection Medicine (CiiM), a joint venture between the Helmholtz Centre for Infection Research (HZI) and Hannover Medical School (MHH), Hannover, Germany; 2 TWINCORE, Centre for Experimental and Clinical Infection Research, a joint venture between the Hannover Medical School and the Helmholtz Centre for Infection Research, Hannover, Germany; 3 Department of Gastroenterology, Hepatology, Infectious Diseases and Endocrinology, Hannover Medical School, Hannover, Germany; 4 Department of Internal Medicine and Radboud Institute for Molecular Life Sciences, Radboud University Medical Center, Nijmegen, Netherlands; 5 Cluster of Excellence RESIST (EXC 2155), Hannover Medical School, Hannover, Germany; 6 Lower Saxony Center for Artificial Intelligence and Causal Methods in Medicine (CAIMed), Hannover, Germany

**Keywords:** single-cell RNA sequencing, hepatocellular carcinoma, systemic immunosuppression, CD14⁺ monocyte-derived gene signature, ABCA1

## Abstract

**Background:**

Liver ablation or resection can cure early-stage hepatocellular carcinoma (HCC), yet late diagnosis and high relapse rates hinder long-term survival. We sought to delineate how tumor burden—and its removal—reshape the systemic immune transcriptome and extract blood-based signatures with diagnostic and prognostic potential.

**Method:**

Peripheral blood mononuclear cells (PBMCs) from six early-stage HCC patients were subjected to single-cell RNA sequencing (scRNA-seq) both prior to and 1–3 months following curative therapy, alongside six age-matched healthy controls. The data were integrated with independent bulk PBMC transcriptomes and public single-cell datasets of paired tumor and adjacent liver immune cells.

**Result:**

Pre-therapy PBMCs displayed an immunosuppressive transcriptional program characterized by elevated TGF beta signaling and ubiquitin-mediated proteolysis. Curative therapy attenuated these pathways and partially restored interferon responses, cytotoxic gene expression, and intercellular communication, although the values remained below healthy levels. We cross-validated these features in tissue, identifying concordant immunosuppressive signatures in tumor versus adjacent-liver immune cells. An immunosuppressive CD14^+^ monocyte subset that expands in the blood of HCC patients displays a transcriptional program matching an IL-10–rich, M2-like macrophage population in liver tissue. A 23-gene signature from this subset was significantly up-regulated in bulk PBMCs from HCC patients (diagnostic) and associated with poor overall survival in the TCGA-LIHC cohort (prognostic). Among these genes, ABCA1 marked monocytes and macrophages with high TGF beta signaling, accurately reflecting tumor-associated immunosuppression in blood and liver.

**Conclusion:**

Early-stage HCC induces a reversible, systemic immunosuppressive transcriptome captured by a monocyte-derived 23-gene blood signature; tumor removal partially restores this profile within three months. These results highlight the potential of blood-derived monocyte signatures as noninvasive biomarkers of HCC-associated immunosuppression and clinical outcome.

## Introduction

Liver cancer, with hepatocellular carcinoma (HCC) constituting approximately 90% of cases, ranks among the most prevalent cancers and is a leading cause of cancer-related mortality worldwide (1). Even after early detection and curative therapy, long-term survival remains poor, and there remains a critical need for reliable blood-based markers for early diagnosis and monitoring of recurrence.

While tumor-oriented research has offered pivotal insights and atlases into local immune responses within the HCC tumor microenvironment (TME) (2–5), the impact of tumor presence on immune cells beyond the TME is less understood. HCC provokes systemic immunosuppression: tumor-conditioned monocytes expand in bone marrow and blood, differentiate into tumor-associated macrophages (TAMs) or myeloid-derived suppressor cells (MDSCs), and circulating cytotoxic T and NK cells become numerically and functionally depleted (6–8). A systems-level understanding of HCC-associated immunosuppression therefore demands concurrent profiling of blood and tissue compartments.

Once a tumor has been ablated, direct tissue analysis is no longer possible, whereas peripheral blood can be sampled serially. Circulating immune cells therefore provide an assessable and informative window into systemic immune changes following tumor removal. Longitudinal comparisons within the same patients—before and after therapy—pinpoint immune programs that are truly tumor driven and reveal whether curative therapy realigns them toward a healthy baseline. Although previous studies have described alterations in circulating immune populations associated with HCC, these analyses were largely limited to bulk RNA sequencing or conventional flow cytometry, lacking the molecular resolution necessary to precisely define specific immune cell subsets (7, 8). Single-cell RNA sequencing (scRNA-seq), in contrast, provides an unbiased, high-resolution view of cellular diversity and transcriptional states at the single-cell level, enabling identification of exact molecular expression markers that distinguish functionally distinct immune subsets (9, 10).

Here, we profiled 26,148 peripheral-blood mononuclear cells (PBMCs) from six early-stage HCC patients sampled immediately before curative therapy and again 1–3 months later, together with age-matched healthy donors. To validate the transcriptional signatures from the discovery group, we integrated our data with public single-cell maps of HCC tumor and adjacent liver tissue, as well as bulk PBMC RNA-seq datasets. Our analysis uncovered a tumor-associated immunosuppressive transcriptional program that extends into the circulation, which is partly attenuated 1–3 months after therapy. A 23-gene immunosuppressive signature derived from the expanded CD14^+^ monocyte subset and anchored by ABCA1, connects blood and liver immune compartments and predicts poor overall survival in TCGA-LIHC cohort. These findings suggest that blood-derived monocyte signatures may provide a noninvasive window into HCC-associated immune dysregulation and support their potential as diagnostic and prognostic biomarkers.

## Methods

### Patient cohort and sample collection

We collected blood samples from six HCC patients with different etiologies for single-cell RNA sequencing. Samples were obtained immediately before—and again one to three months after—curative therapy (radiofrequency ablation, n = 5; surgical resection, n = 1) between December 2021 and June 2022. Although our cohort includes predominantly ablation and only one surgical resection, the direction and magnitude of immune and inflammatory response changes were highly concordant across both therapy approaches (**Supplementary Figure S1E**). All patients were serologically screened for viral hepatitis; two were HCV‐positive, and none had received chemotherapy or other anticancer treatments. Detailed information about the patient characteristics is listed in **Supplementary Figure S1A**. Age-matched healthy donors (n = 6; mean age 61.6 years) were recruited from our previously published cohort (11) to serve as controls (HCC patients mean age 67.5 years). PBMCs were isolated by standard density gradient centrifugation from whole blood using Pancoll (PAN BIOTECH, Aidenbach, Germany) following the manufacturer’s instructions at Hannover Medical School. The isolated cells were cryopreserved until use.

### Ethics statement

This study was conducted in accordance with the principles of the 2013 Declaration of Helsinki and the 2018 Declaration of Istanbul. All patients provided written informed consent to the study. The ethics committee of Hannover Medical School approved the protocols, including blood sample collection (no._9474_BO_K_2020, no._940-2011, no._10045_BO_K_2021, and no._172/06).

### Single-cell RNA-seq processing and quality control

We processed sequencing data using Cell Ranger v6.1.0 (10X Genomics), aligned reads to the human genome reference GRCh38 V2020-A and generated a gene-cell count matrix for each library. DNA from PBMC samples was genotyped using the Infinium Global Screening Array Kit (Illumina), and genotypes were called using OptiCall (v0.7.0). Using Souporcell (12) (v2.0), we assigned cells to original donors and removed duplicates. Cells with fewer than 500 or more than 6,000 features, or more than 25,000 gene counts, or more than 10% mitochondrial reads, or more than 40% ribosomal content were excluded, resulting in 26,148 high-quality cells with 25,438 features for downstream analysis (**Supplementary Figure S1B**).

### Public data sources

Single-cell RNA-seq data from HCC tumor tissue were obtained from GEO (GSE149614) and the China National GeneBank (CNP0000650). Tissue-derived immune cells were extracted and filtered using the same thresholds described above. Bulk PBMC RNA-seq datasets were obtained from a previously published study (13) and the GEO (GSE58208) for external validation.

### Data integration and clustering

We processed and integrated all single‐cell datasets in Seurat (v5.0.0) (14) to mitigate batch effects. Each dataset was normalized (scale factor = 10,000) and the top 2,000 variable features selected prior to running SelectIntegrationFeatures(), FindIntegrationAnchors(), and IntegrateData(). The merged data were scaled and subjected to PCA, retaining the first 20 PCs. We constructed an SNN graph (k = 20) and computed UMAP embeddings, clustering at a resolution of 0.5. Cell types were annotated using Azimuth reference signatures (15). For sub‐clustering, PBMC monocytes, PBMC lymphocytes, and tissue‐derived monocytes were each re‐analyzed: PBMC subsets were clustered at resolution 0.2, while tissue monocytes used resolution 0.5.

### Differential expression and pathway enrichment analysis

We performed differential expression analysis using the FindMarkers function in Seurat (test.use = “MAST”), specifying patient ID as a latent variable to count for repeated measures. DEGs were defined as genes detected in ≥10% of cells with |log_2_FC| > 0.25 and p_val_adjusted < 0.05. Pathway enrichment analysis utilized Hallmarks, Gene Ontology, and Reactome pathways from MSigDB (16).

### Identifying differential gene expression modules

We clustered DEG log-fold change profiles using k-means to capture differences in cell populations before and after therapy. Before clustering, non-significant values (p > 0.05) were set to zero. Ontology enrichment was performed using clusterProfiler (v3.16.1) (17) on clustered heatmap modules, selecting the most statistically significant ontologies for presentation.

### Gene signature enrichment

We used UCell (v2.0.1) (18) to calculate area-under-the‐curve (AUC)–based enrichment scores for each cell. Pathways included HALLMARK INTERFERON ALPHA RESPONSE, HALLMARK INTERFERON GAMMA RESPONSE, HALLMARK TGF BETA SIGNALING, HALLMARK INFLAMMATORY RESPONSE, Activation of immune response (GO), Leukocyte mediated immunity (Reactome), Ubiquitin mediated proteolysis (Kegg), macro-autophagy (GO), and interleukin-10 signaling (Reactome) were from MSigDB. Interferon scores were computed using 57 genes from Modules 4 and 5. Cytotoxicity scores were calculated using expression of PRF1, IFNG, GNLY, NKG7, GZMB, GZMA, CST7, GZMK, and TNFSF10 (3). The genes used to define M1/M2 polarization and pro-inflammatory states in monocyte subsets are listed in **Supplementary Table S2D**.

### Cell-cell communication analysis

Intercellular communication analysis was performed using CellChat (v2.1.2) (19) on both PBMC and tumor tissue data. Secreted signaling interactions were inferred for each condition. CellChat identified over-expressed ligands and receptors for each cell type, and used joint manifold learning classified networks based on functional and topological similarities across conditions. Differential expression analysis identified upregulated and downregulated ligand-receptor pairs based on fold changes of ligands in sender cells and receptors in receiver cells, using a ligand logFC threshold of ≥0.1. The intercellular communication network was visualized using the circlize R package (v0.4.13).

### Differential cell-type abundance analysis

We performed differential abundance analysis pre- vs. post-therapy on a WNN graph using miloR (v1.4.0) (20). The wsnn graph served as input for the buildFromAdjacency function (k = 30, d = 30). We assigned cell neighborhoods with makeNhoods (prop = 0.1, refinement = ‘graph’) and quantified neighborhood cell counts using countCells. The testNhoods function (design = ~tumor_curative_therapy, fdr.weighting = ‘graph-overlap’) identified neighborhoods with SpatialFDR < 0.1 as differentially abundant.

### Cell developmental trajectory

We constructed cell lineage trajectories using monocle3 (v1.3.7) (21). A CellDataSet object was created from the Seurat object via the as.cell_data_set () function. We then performed dimensionality reduction with reduce_dimension().

### TCGA data analysis

Gene expression and clinical data for TCGA-LIHC were retrieved using TCGAbiolinks (v2.24.3). A 23-gene CD14^+^ mono3 signature and single gene expression were quantified as the mean log_2_-expression per sample. To account for immune cell content, scores were normalized to PTPRC (CD45) expression. Kaplan–Meier survival curves were stratified by median adjusted signature values, and differences assessed via log-rank test. Gene–gene correlations were computed using Spearman’s method. Multivariable Cox proportional hazards regression was performed using the coxph() function from the survival (v3.8.3), adjusting for age and sex. A two-sided P < 0.05 was considered significant.

### Bulk PBMC RNA-seq analysis

Raw count matrices were pre-processed by filtering genes with low expression, retaining those with rowSums (countData ≥ 10) ≥ 5. Differential expression analysis was conducted using DESeq2, with thresholds set at |log_2_FC| > 0.25 and p_val_adjusted < 0.05. For ROC analysis, count data were log_2_-transformed using log_2_ (count + 1), and average expression of the 23-gene signature was calculated per sample. ROC curves were generated using the pROC (v1.19.0.1).

### Defining ABCA1^+^ and ABCA1^−^ myeloid subsets

In PBMCs and our primary liver-tissue dataset, monocytes or macrophages were classified as ABCA1^+^ if their normalized ABCA1 expression exceeded zero and ABCA1^−^ if it was exactly zero (**Supplementary Figures S5A, D**). For the independent tumor cohort—where almost no cells had zero expression—we instead designated cells with ABCA1 expression ≥ 1 as ABCA1^+^ and those with expression < 1 as ABCA1^−^ (**Supplementary Figure S5E**). These ABCA1^+^ and ABCA1^−^ subpopulations were used for downstream differential expression, pathway enrichment, and signature scoring analyses.

### Statistical analysis

Cell‐type proportions were compared by two‐tailed Wilcoxon tests: paired for pre- vs. post-therapy and unpaired for healthy controls vs. pre- or post-therapy. Comparisons of gene expression or gene signatures between two cell groups were performed with an unpaired two-tailed Wilcoxon rank sum test. Genome-wide differential expression and pathway enrichment analyses were performed using appropriate models, and p-values were adjusted for multiple testing using the Benjamini-Hochberg false discovery rate (FDR) method. Unadjusted p-values are reported in exploratory comparisons. All analyses and visualizations were done in R (v4.2.0). Full statistical details are provided in the respective figure legends, which specify whether p-values are adjusted or unadjusted.

## Results

### Single-cell transcriptional landscape of peripheral blood immune cells in HCC patients undergoing curative therapy

**Figure 1:**
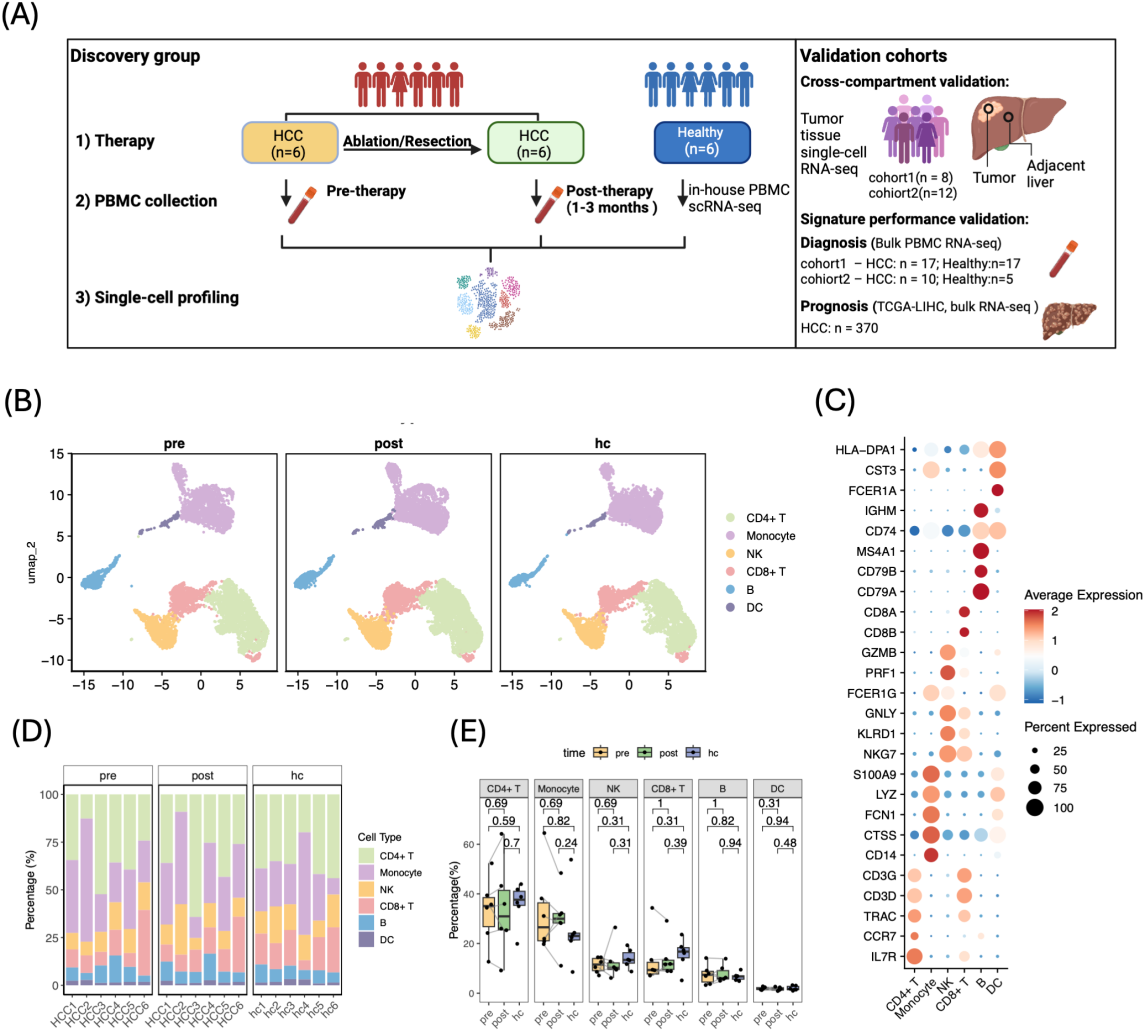
Single‐cell transcriptomic landscape of peripheral blood immune cells in HCC patients. **(A)** Study overview. Created with BioRender.com. **(B)** UMAP projections of PBMCs from pre‐therapy (pre), post‐therapy (post), and healthy control (hc) samples, colored by six major immune lineages. **(C)** Dot plot of canonical marker genes used for cell‐type annotation; dot size indicates percentage of cells expressing each gene, color scale represents average expression. **(D)** Stacked bar plots showing immune cell composition per patient in the three groups. **(E)** Box plots comparing the frequency of each major lineage across conditions; medians indicated by horizontal bars, whiskers represent interquartile range. P values were calculated using paired two-tailed Wilcoxon test (pre vs. post) or unpaired two-tailed Wilcoxon tests (hc vs. pre/post), as indicated.

To reveal systemic immune changes elicited by tumor removal, we performed scRNA-seq on PBMCs collected from six early-stage HCC patients, alongside six age-matched healthy donors. Each patient provided paired samples taken immediately before ablation or resection and again 1–3 months afterwards, a timeframe chosen to capture long-term immune adaptation after acute post-surgery inflammation subsided (22) (**Figure 1A**). Clinical characteristics, including liver disease status, fibrosis/cirrhosis, liver function, and tumor size, are summarized in **Supplementary Figure S1A**.

To verify our blood-based transcriptional signatures and test their consistency between circulating and tissue-resident immune cells, we analyzed three independent datasets: two scRNA-seq cohorts comprising matched tumor and the adjacent-liver tissue from 20 HCC patients (3, 5), and two bulk PBMC RNA-seq cohorts including 17 HCC patients and 17 healthy donors (13), and 10 HCC patients with 5 healthy donors. In addition, we assessed the prognostic value of the transcriptional signatures in the TCGA-LIHC cohort (**Figure 1A**, external comparison cohorts).

After quality control and removal of low-quality cells, we retained 26,148 high-quality cells (6,701 pre-therapy,10,015 post-therapy and 9,432 healthy) for downstream analysis (**Supplementary Figure S1B**). Unsupervised clustering resolved six major immune cell types, annotated with established marker sets (15) (**Figures 1B, C**). All subsets were detected in every individual, but their relative proportions varied substantially across patients, consistent with known heterogeneity of tumor-infiltrating immune cells among HCC tumors (3) (**Figure 1D**). Importantly, paired comparison revealed no global shift in the frequencies of these broad lineages after therapy (**Figure 1E**), suggesting a stable peripheral immune composition under curative therapy for HCC patients.

### Curative therapy partially reverses tumor-associated transcriptional suppression in peripheral blood

**Figure 2:**
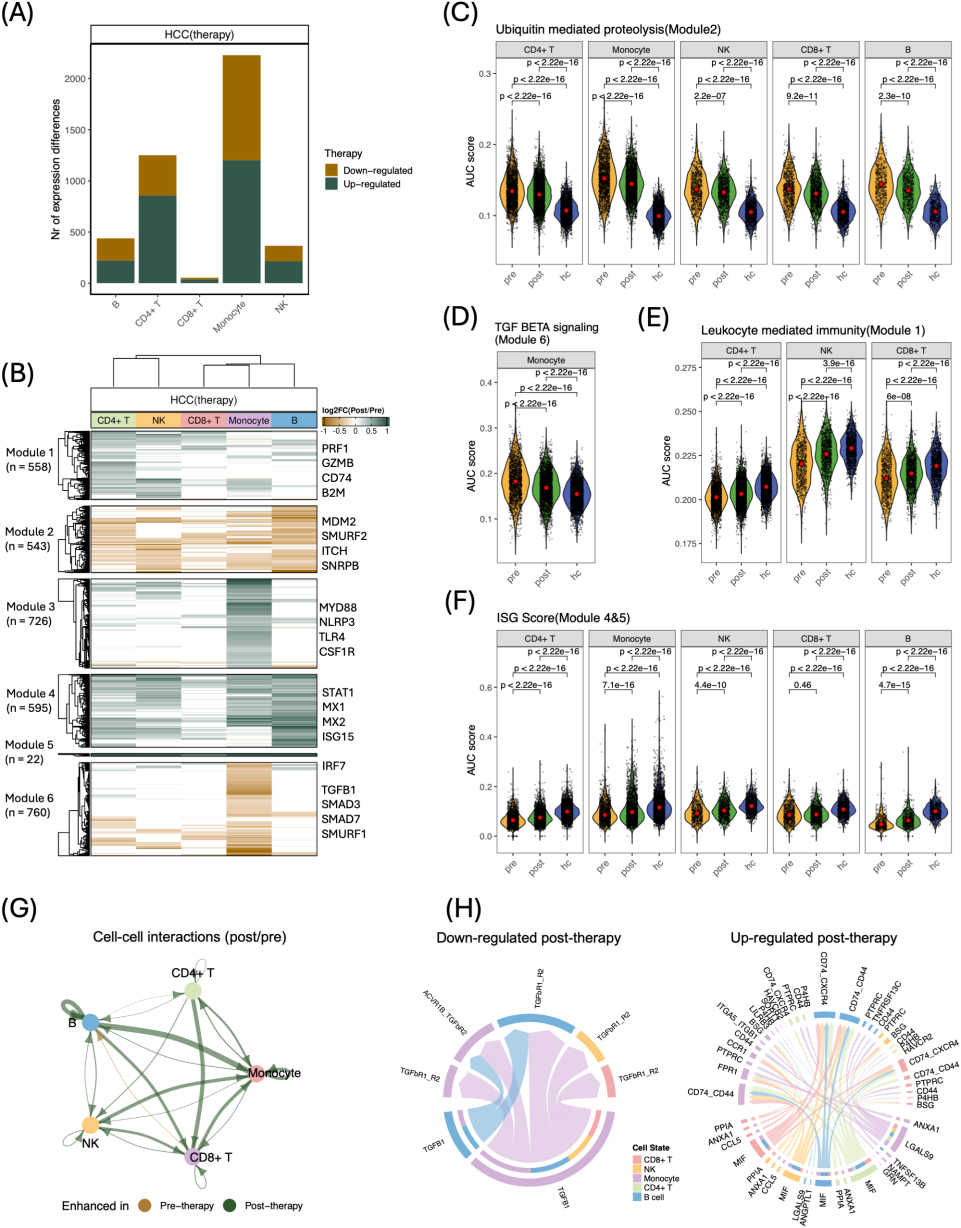
Transcriptional and intercellular remodeling of PBMCs following curative therapy. **(A)** Number of differentially expressed genes (DEGs) per lineage (FDR < 0.05, |log_2_ FC| > 0.25); brown bars indicate genes down-regulated post-therapy, green bars indicate up-regulated genes. **(B)** Unsupervised clustering of 3,222 DEGs identifies six co-expression modules; heatmap shows centered log_2_ FC across lineages (brown = up, green = down, white = no change). **(C–F)** Violin plots of AUC-based signature scores across pre, post, and hc conditions; red dots denote medians, P values calculated by unpaired two-tailed Wilcoxon tests. **(C)** Ubiquitin-mediated proteolysis. **(D)** TGF-β signaling in monocytes. **(E)** Leukocyte-mediated immunity in T and NK cells. **(F)** Interferon-stimulated gene (ISG) signature derived from Modules 4 & 5. **(G)** Differential cell–cell interaction network (post vs. pre); edge width reflects change in aggregate signaling (green = increased post, brown = increased pre). **(H)** Circos plots of ligand–receptor interactions down-regulated (left) and up-regulated (right) post-therapy; ribbon width represents interaction strength.

Given the stable immune-cell composition after therapy, we next investigated whether deeper functional transcriptional changes revealed immune consequences of tumor removal. Paired differential-expression analysis identified 3,222 differentially expressed genes (DEGs) across the major immune compartments (**Figure 2A**; false discovery rate (FDR)-adjusted p < 0.05, |log2FC| > 0.25; **Supplementary Table S1A**). Monocytes showed the highest number of DEGs (2,228), indicating their central role in systemic immune adaptations following tumor removal.

To comprehensively analyze gene expression dynamics during therapy, we grouped all DEGs into six modules via unsupervised clustering, followed by functional enrichment analysis (**Figure 2B**, **Supplementary Table S1B**). Module 2, downregulated across lineages after therapy, was enriched for Ubiquitin mediated proteolysis. Ubiquitination affects immune cell function and contributes to immune suppression in the tumor microenvironment (23). To further examine this pathway activity during therapy relative to healthy donors, we applied UCell (18) to compute an area-under-the‐curve (AUC)-based enrichment score for all 136 pathway genes. The results revealed significantly reduced activity after therapy, though levels remained elevated compared to healthy donors (**Figure 2C**). Module 6, decreased mainly in monocytes, was enriched for TGF Beta signaling, featuring master regulator TGFB1 and key transcriptional regulator SMAD3. TGF-β serves a pivotal role in regulating tumor-infiltrate immune cells, thereby driving potent immunosuppressive activity in HCC (24). Signature scoring of the 54-gene set confirmed a significant post-therapy reduction, yet activity remained above control values (**Figure 2D**). These findings suggest that tumor-associated peripheral immune transcriptional changes involve ubiquitin-related pathways across immune cell types and TGF-β signaling specifically in monocytes.

We then examined modules that increased after therapy. Module 1, elevated in T and NK cells, was enriched for Leukocyte mediated immunity and contained cytotoxic genes such as PRF1 and GZMB; the associated 470-gene cytotoxicity score increased post-therapy but did not reach healthy-donor levels (**Figure 2E**). In line with this activated profile, the monocyte-specific Module 3 was enriched for genes involved in the positive regulation of cytokine production, indicating enhanced cytokine output after therapy. Module 4 and module 5 were broadly upregulated after therapy, enriched for Interferon alpha and gamma response and included canonical IFN-induced genes—STAT1, IRF7, ISG15, and MX2. An interferon-stimulated gene (ISG) score, derived from 57 ISGs identified from these modules, demonstrated significantly increased IFN activity across all cell types after therapy in HCC patients, approaching but not exceeding healthy level (**Figure 2F**). These findings support an association between tumor removal and enhanced cytotoxicity, cytokine production, and interferon signaling.

Taken together, our results reveal a systemic transcriptional program characterized by elevated ubiquitin and TGF-β signaling during tumor presence that is partially alleviated 3 months after curative therapy, with monocytes exhibiting the greatest responsiveness to these changes.

### Enhanced cell-cell interactions reflect immune activation after curative therapy

To investigate whether the transcriptional recovery observed post-therapy was paralleled by changes in intercellular signaling, we performed cell-cell communication analysis using CellChat (19). This approach revealed a general increase in predicted immune cell interactions following therapy (**Figure 2G**, **Supplementary Figures S1C, D**), suggesting that tumor removal strengthens peripheral immune communication networks. Notably, TGF-β-mediated interactions (TGFB1-TGFbR1/TGFbR2/ACVR1B) predominantly from monocytes to B, CD8^^+^^ T and NK cells, were significantly reduced after therapy. Conversely, the most notable upregulated signaling involved MIF (MIF-CD74/CXCR4) and GALECTIN (LGALS9-PTPRC/CD44/P4HB) (**Figure 2H**). MIF (Macrophage migration inhibitory factor) exerts essential proinflammatory roles in both monocytes and T cells (25), and LGALS9 (Galectin-9) can activate proinflammatory genes in monocytes and promote Th1 immune responses (26). These results suggest that curative therapy is associated with reduced TGF-β–related inhibitory crosstalk and increased proinflammatory communication among peripheral immune cells.

### Shift from tumor-associated suppression to a pro-inflammatory phenotype in peripheral monocytes following curative therapy

**Figure 3:**
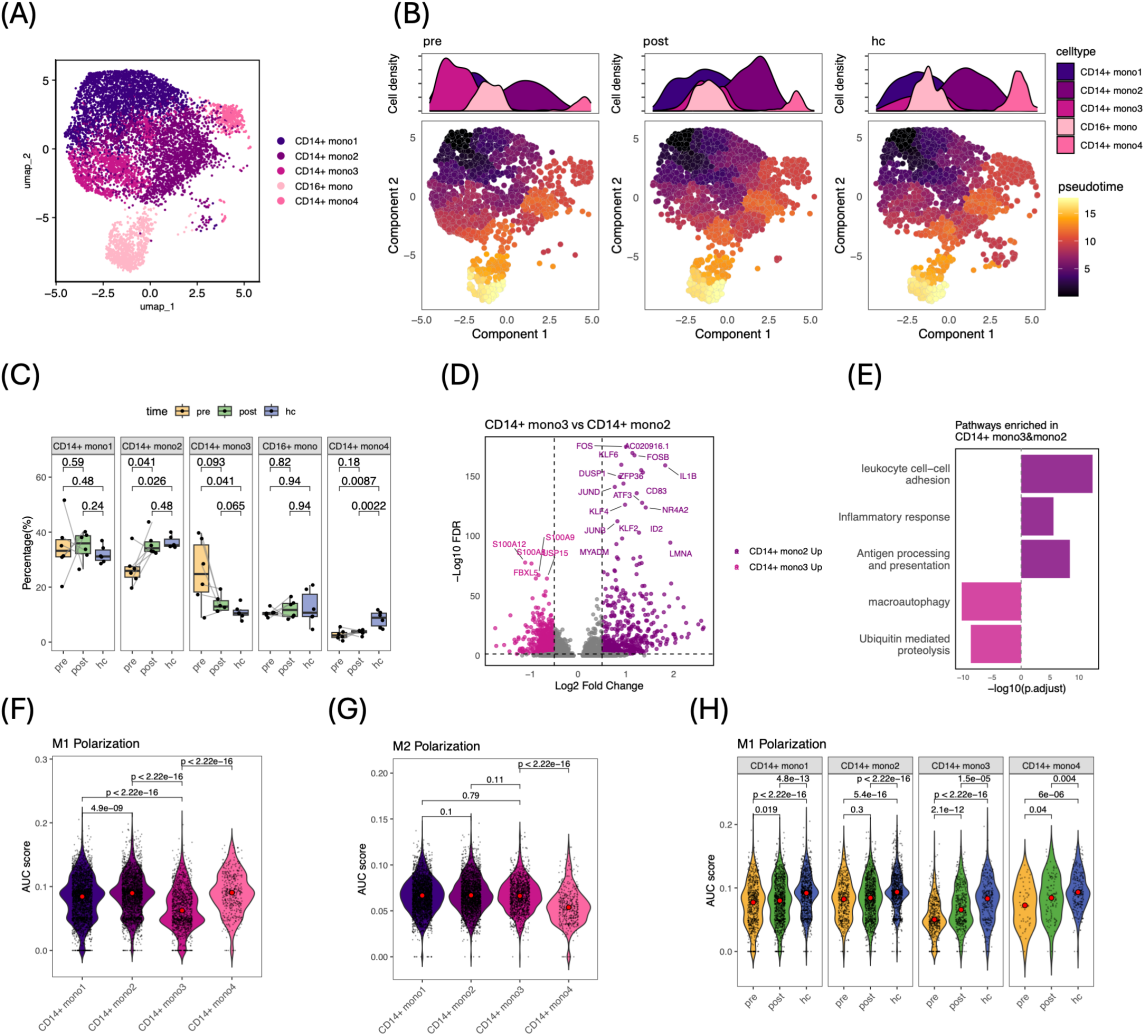
Dynamic transitions and functional reprogramming of circulating monocytes following curative therapy. **(A)** UMAP of PBMC-derived monocytes from HCC patients and healthy donors, identifying five subsets: CD14^+^ mono1–mono4 and CD16^+^ mono. **(B)** Trajectory analysis with cells colored by subset (left) and by pseudotime (right); bottom: pseudotime color scale; top: subset density along trajectory. **(C)** Box plots of monocyte subset frequencies across pre, post, and hc groups; medians indicated by bars, whiskers represent interquartile range. P values were calculated using paired two-tailed Wilcoxon test (pre vs. post) or unpaired two-tailed Wilcoxon tests (hc vs. pre/post), as indicated. **(D)** Volcano plot of DEGs between CD14^+^ mono2 and CD14^+^ mono3 (FDR < 0.05, |log_2_ FC| > 0.25). **(E)** Pathway enrichment highlighting functional programs distinguishing mono2 and mono3. **(F, G)** Violin plots of AUC-based polarization signature scores across CD14^+^ subsets: **(F)** M1 and **(G)** M2 signatures. **(H)** Violin plots of M1 signature scores in mono subsets under pre, post, and hc conditions; red dots = medians. P values calculated using unpaired two-tailed Wilcoxon tests.

Monocytes are of particular interest because they are the major precursors of TAMs and MDSCs in circulation (27), prompting us to focus on characterizing these cells. Five monocyte subtypes formed discrete clusters on the UMAP (**Figure 3A**, **Supplementary Figure S2A**). Next, we investigated how therapy influences the dynamic immune states and transitions of monocytes using monocle3 (21). This analysis placed CD14^+^ mono1 at the root, bifurcating into the divergent CD14^+^ mono2 and mono3 states before converging on CD16^+^ monocytes (**Figure 3B**, **Supplementary Figure S2B**). Cells from the pre-therapy samples were enriched along the mono3 branch, whereas post-therapy and healthy-donor cells accumulated preferentially in the mono2 branch, suggesting that tumor removal is associated with a shift in monocyte maturation toward patterns observed in healthy donors. Consistent with this shift, pre-therapy samples exhibited a significantly lower proportion of CD14^+^ mono2 cells (p = 0.02) and a higher proportion of CD14^+^ mono3 cells (p = 0.04) compared with healthy donors. By contrast, post-therapy monocyte subset frequencies did not differ significantly from healthy controls, indicating partial normalization of the peripheral monocyte immune states (**Figure 3C**). CD14^+^ mono4 cells were relatively depleted in patients at both time-points. Per-sample inspection revealed no outliers and greater uniformity post-therapy, indicating a consistent therapeutic impact (**Supplementary Figure S2C**).

Differential-expression analysis between mono2 and mono3 (FDR < 0.05, |log_2_FC| > 0.25) identified higher expression of pro-inflammatory genes (FOS, FOSB, IL1B) in mono2 (**Figure 3D**). Pathway and gene signature enrichment analysis confirmed that mono2 was enriched for antigen presentation and inflammatory-response pathways, whereas mono3 was predominantly associated with ubiquitin-mediated proteolysis and macro-autophagy (**Figure 3E**; **Supplementary Figure S2D**; **Supplementary Table S2A**). Pathway activity scores, including TGF-β signaling, partially reverted toward healthy donor levels 1–3 months post-therapy, with different magnitude across monocyte subsets (**Supplementary Figures S2E–H**).

To examine whether tumor presence influences peripheral monocyte differentiation to anti-inflammatory phenotype, we evaluated unique monocytes states by calculating M1 and M2 polarization scores (3). Notably, mono2 exhibited the highest M1 polarization score, while mono3 displayed the lowest, with minimal variation in M2 polarization score (**Figures 3F, G**). Importantly, the M1 score of mono3 increased significantly following therapy, with a mean shift from 0.054 to 0.069, approaching but still below the range of healthy donors (mean = 0.08; **Figure 3H**). Thus, during tumor presence, circulating monocytes display reduced M1−like features without strong induction of classical M2 programs, and curative therapy partially restores the pro-inflammatory state. Finally, the healthy-restricted mono4 subset displayed both the lowest M2 score (**Figure 3G**) and the weakest oxidative-phosphorylation signature (**Supplementary Figure 2I**), suggesting metabolic changes in the HCC patients and not yet regained post-therapy.

Collectively, these findings reveal a transcriptional program in peripheral monocytes consistent with systemic immune suppression before therapy, and curative therapy partially reprograms them toward a more pro-inflammatory phenotype.

### Immunosuppressive lymphocyte states dominate before curative therapy

Because lymphocytes play a pivotal anti-tumor role in HCC^1^, we resolved their heterogeneity by lineage-specific clustering, identifying four CD4^+^ T-cell states, three CD8^+^ T-cell states, two NK subsets and three B-cell subsets (**Supplementary Figures 3A, B**). Differential-abundance test using miloR (20) detected 14 cellular neighborhoods significantly enriched in pre-therapy samples (FDR < 0.1; **Supplementary Figure 3C**). Most of these corresponded to CD4^+^ naïve, CD4^+^ TCM and NK CD56dim cells, whose gene signatures were under-enriched for leukocyte activation and immune-response pathways (**Supplementary Figures S3D, E**; **Supplementary Table S2B**), indicating a tumor-associated expansion of quiescent or immunosuppressive lymphocyte populations.

A distinct “B-unknown” subset was also enriched pre-therapy samples. Compared to other B cell populations, this subset showed downregulation of CD79B, MS4A1, and HLA-E (**Supplementary Figure S3F**). Pathway enrichment analysis indicated reduced B cell activation and elevated activity of ubiquitin-mediated proteolysis pathways – features consistent with an immunosuppressive phenotype (**Supplementary Figures 3F, G**; **Supplementary Table S2C**).

Following tumor removal, the lymphocyte compartment shifted toward a more activated state. The frequency of CD8^+^ TEM cells increased (**Supplementary Figure 3H**), and cytotoxicity scores rose across effector CD4^+^, CD8^+^ and NK cells, approaching levels observed in healthy donors (**Supplementary Figure 3I**). Together, these findings support the presence of a systemic low−activation state before therapy that is only partially reversed after curative therapy.

### Tumor-imposed suppression and impaired cell–cell interactions in liver tissue

**Figure 4:**
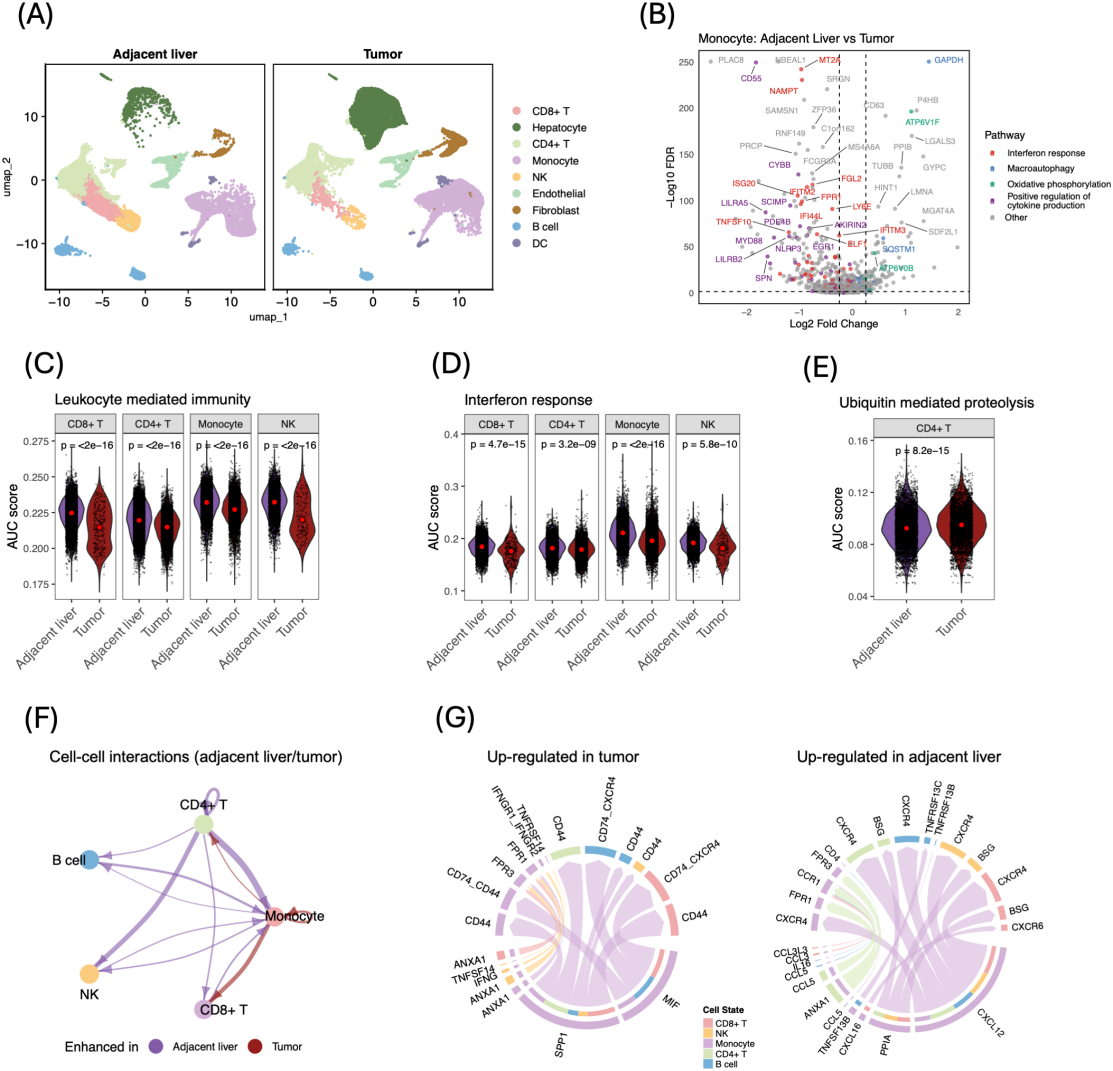
Immune cell composition, transcriptional programs, and intercellular signaling in tumor versus adjacent-liver tissue. **(A)** UMAP projections of immune cells from matched tumor and adjacent-liver samples (n = 8 patients), colored by major lineage. **(B)** Volcano plot of monocyte DEGs (tumor vs. adjacent liver; FDR < 0.05, |log_2_ FC| > 0.25), with key genes highlighted by pathway. **(C–E)** Violin plots of AUC-based signature scores comparing adjacent-liver versus tumor samples; red dots = medians. P values calculated using unpaired two-tailed Wilcoxon tests. **(C)** Leukocyte-mediated immunity. **(D)** Interferon-response. **(E)** Ubiquitin-mediated proteolysis. **(F)** Cell–cell interaction network (adjacent liver vs. tumor); edge width = interaction magnitude (purple = stronger in liver, red = stronger in tumor). **(G)** Circos plots of up-regulated ligand–receptor interactions in tumor (left) and adjacent liver (right); ribbon width = interaction strength.

To test whether the systemic immunosuppression features identified in peripheral blood originate in, or coexist with, the tumor microenvironment, we analyzed a public scRNA-seq dataset comprising paired tumor and adjacent non-tumor liver tissue from eight patients with HCC (5). After quality control, 50, 436 cells were retained, including 35, 741 immune cells. Using the same marker set employed in our PBMC analysis, we annotated all major immune cell types (**Figure 4A**, **Supplementary Figures 4A, B**). As reported previously in these cohorts, tumors contained more monocytes and fewer CD8^+^ T and NK cells than adjacent liver (3, 5) (**Supplementary Figures 4C, D**).

We asked whether the systemic immunosuppressive imprint observed in pre-therapy PBMCs is enriched in tumor-infiltrating immune cells relative to adjacent, non-tumor liver tissue. To address this, we cross-validated the PBMC DEGs by analyzing matched tumor and adjacent-liver immune cells on a per-cell-type basis. Among the genes that increased in PBMCs after therapy, 567 were likewise up-regulated in adjacent liver, whereas 319 genes elevated in pre-therapy blood were preferentially expressed in tumor immune cells; of which the majority (598 genes) were detected in monocytes (**Supplementary Table S3A**). Differential expression analysis revealed that adjacent-liver monocytes up-regulated genes linked to interferon response and cytokine production compared with tumor-infiltrating monocytes, whereas tumor-infiltrating monocytes showed higher expression of genes involved in oxidative phosphorylation and macroautophagy (**Figure 4B**; **Supplementary Table S3B**). Strikingly, 32 out of 54 interferon-response genes elevated in adjacent-liver monocytes corresponded to the module 4&5–gene modules that were upregulated in the peripheral blood post-therapy (**Figure 2F**). Parallel patterns emerged in other immune lineages: cells from adjacent liver exhibited significantly stronger interferon responses, antigen-presentation programs, and receptor-signaling activity than their tumor-infiltrating counterparts (**Supplementary Table S3C**).Signature scoring using PBMC-derived gene sets further confirmed these findings. Tumor-infiltrating immune cells showed reduced leukocyte-mediated immunity and interferon activity relative to those in adjacent liver (**Figures 4C, D**). Conversely, ubiquitin-mediated proteolysis was selectively elevated in tumor CD4^+^ T cells (**Figure 4E**). Interestingly, TGF-β signaling was enriched in monocytes from adjacent liver tissue (**Supplementary Figure S4F**), consistent with the known homeostatic role of TGF-β in non-tumor liver tissue (28).

Cell-cell communication analysis reinforced the dichotomy. Predicted intercellular interaction strength was markedly reduced in tumor tissue compared to adjacent liver tissue (**Figure 4F**, **Supplementary Figure S4G**). Within tumors, immune communication networks were dominated by monocyte-derived MIF (MIF–CD74/CXCR4) and SPP1 (SPP1–PTPRC/CD44/P4HB) signaling pathways, mediating interaction with other immune cells (**Figure 4G**). Notably, the SPP1–CD44 axis has been implicated in the recruitment of TAMs and the suppression of T and NK cell activity, while MIF, although transitionally viewed as pro-inflammatory, can promote immune evasion and TAMs- mediated tumor migration and tumor progression by acting upstream of SPP1 (29, 30). In contrast, immune cells in adjacent liver primarily engaged in communication via CXCL (CXCL12-CXCR4) and PPIA (PPIA-BSG) signaling pathways, both of which are associated with enhanced leukocyte migration and survival (31, 32) (**Figure 4G**). The prominence of these pathways suggests heightened migratory activity and immune responsiveness in the adjacent liver microenvironment relative to the tumor.

Collectively, these tissue data validate and extend our peripheral blood findings, showing that HCC elicits parallel transcriptional and intercellular immunosuppressive programs both locally within the tumor microenvironment and systemically in circulation.

### Peripheral immunosuppressive gene signature derived from CD14^+^ mono3 cells aligns with an M2-like, IL-10–rich macrophage subset in liver tissue

**Figure 5:**
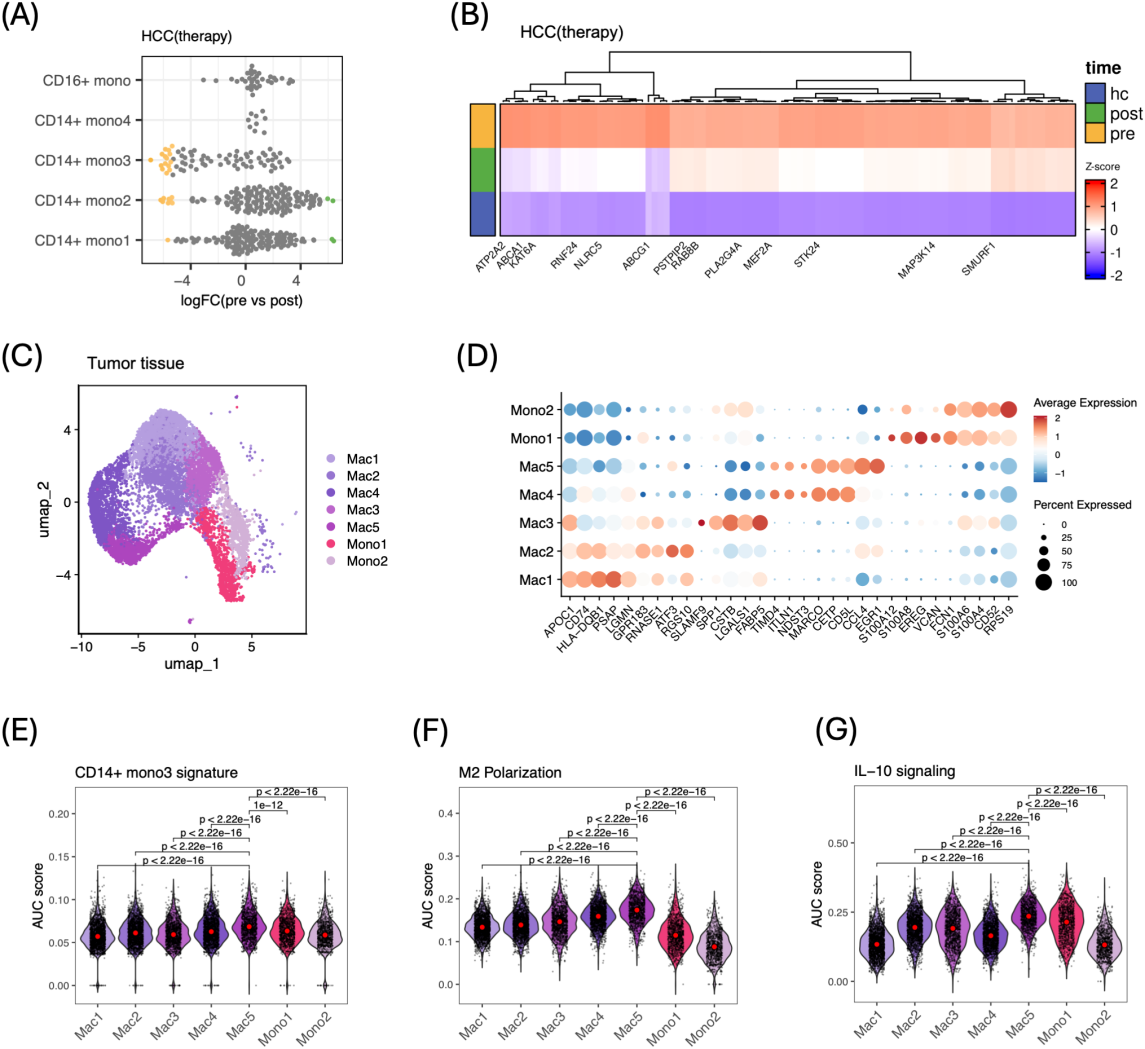
Linking circulating mono3 cells to immunosuppressive macrophages in tumor tissue. **(A)** MiloR differential abundance analysis; each point is a KNN neighborhood, X-axis = log_2_ FC (pre vs. post), Y-axis = –log_10_ FDR, yellow points = enriched pre-abundant neighborhoods. **(B)** Heat map of the 95 highest-loading mono3 genes (z-score) across pre, post, and hc samples. **(C)** UMAP of tumor myeloid cells identifying five macrophage subsets (Mac1–Mac5) and two monocyte subsets (Mono1–Mono2). **(D)** Dot plot of representative markers by subset; dot size = expression frequency, color = average expression. **(E)** Violin plot of AUC-based CD14^+^ mono3 signature scores across tumor subsets; Mac5 is most enriched. **(F, G)** Violin plots of AUC-based M2 polarization **(F)** and IL-10 signaling **(G)** scores across subsets; red dots = medians. P values calculated using unpaired two-tailed Wilcoxon tests.

Given the abundance of CD14^+^ mono3 cells prior to tumor removal and their pronounced immunosuppressive phenotype, we derived a peripheral immunosuppressive gene signature (CD14^+^ mono3 signature) consisting of 95 genes from MiloR-defined neighborhoods significantly enriched pre-therapy (**Figure 5A**; **Supplementary Table S3D**). Visualizing the z-scored expression of these genes across pre-, post-therapy, and healthy control monocytes revealed a clear gradient: highest expression before therapy, lower expression after therapy, and lowest expression in healthy donors (**Figure 5B**).

To determine whether this peripheral immunosuppressive program is recapitulated within the liver tissue, we applied the CD14^+^ mono3 signature to monocytes/macrophages from tumor and adjacent-liver tissue. “Mono3-AUC^high^” macrophages showed significantly higher M2-polarization scores and TGF-β signaling than “Mono3AUC^low^” cells in both tumor and adjacent liver (**Supplementary Figures S4H, I**). Unsupervised clustering further identified five macrophage populations (Mac1–Mac5) and two subsets resembling circulating monocyte (Mono1, Mono2) (**Figures 5C, D**). Scoring individual cells with the CD14^+^ mono3 signature revealed a pronounced enrichment in the Mac5 population (**Figure 5E**).

Consistent with the established role of tumors in driving monocyte-to-TAM differentiation that promotes local immunosuppression (8), Mac5 was positioned at the immunoregulatory end of the functional spectrum. It exhibited the highest M2 polarization score among all subsets (**Figure 5F**). Consistent with the defining property of M2 macrophages—their capacity to secrete IL-10 (33)—Mac5 also displayed the strongest IL-10 signaling activity (**Figure 5G**; **Supplementary Figure S4J**).

Collectively, these results indicate that the immunosuppressive CD14^+^ mono3-derived signatures observed in peripheral blood is recapitulated in an IL-10–rich, M2-like macrophage subset within the tissue, suggesting shared transcriptional programming across systemic and hepatic myeloid compartments.

### Bulk transcriptomic validation highlights clinical relevance of the CD14^+^ mono3-derived signature and identifies ABCA1 as a tractable biomarker

**Figure 6:**
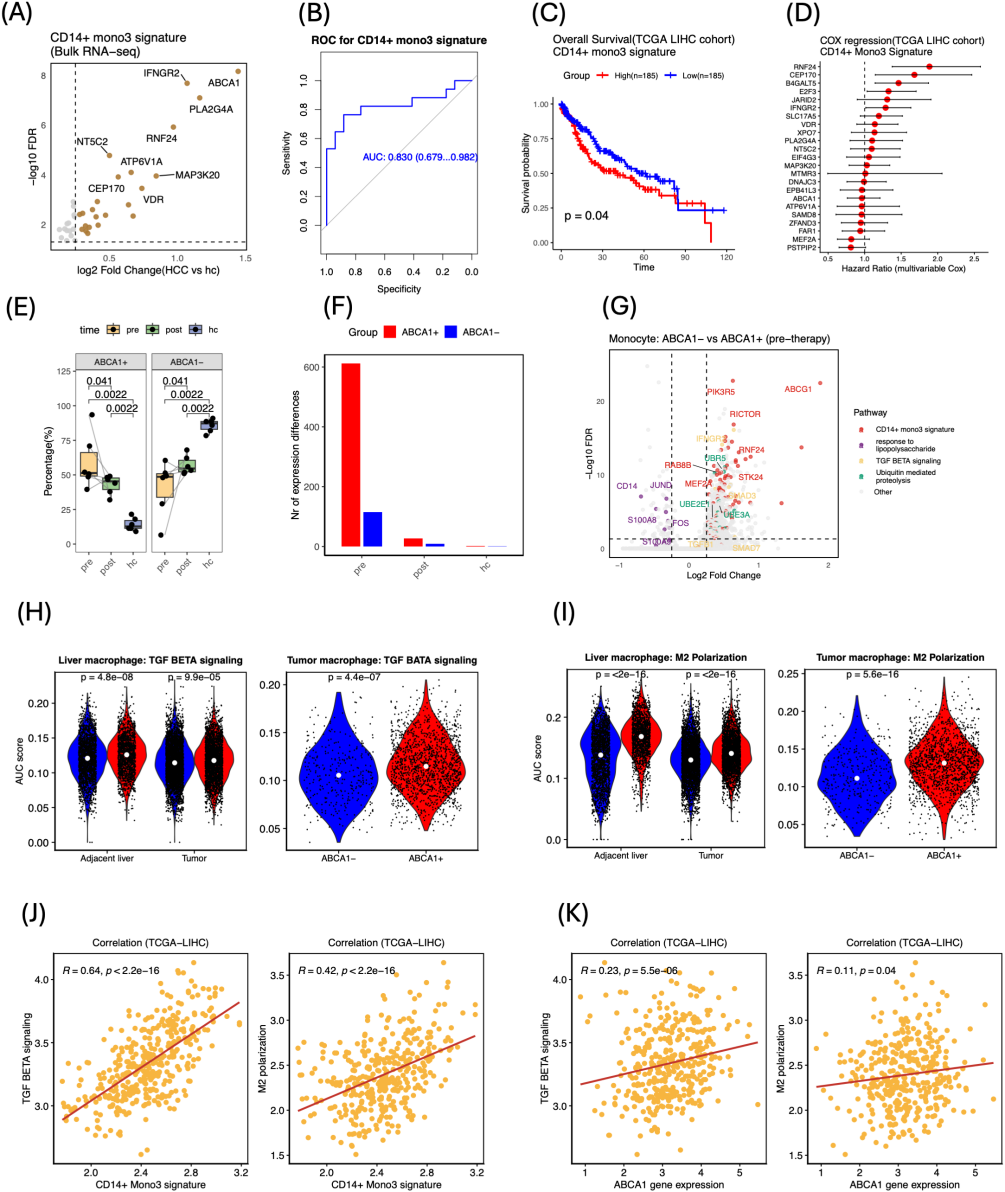
Bulk transcriptomic validation and clinical relevance of the mono3 program and ABCA1^+^ myeloid cells. **(A)** Bulk PBMC RNA-seq (17 HCC vs. 17 HC) volcano plot of 23-gene signature up-regulated in HCC (FDR < 0.05, log_2_ FC > 0.25). **(B)** ROC curve demonstrating diagnostic performance of the 23-gene mono3 signature. **(C)** Kaplan–Meier survival curves (TCGA-LIHC): high vs. low mono3 signature, P values = log-rank test. **(D)** Forest plot of multivariable Cox regression showing hazard ratios for the 23-gene mono3 signature and its individual genes. **(E)** Frequencies of ABCA1^+^ vs. ABCA1^−^ monocytes across pre, post, and hc samples. P values calculated using unpaired two-tailed Wilcoxon tests. **(F)** Number of DEGs between ABCA1^+^ and ABCA1^−^ monocytes at each time point (FDR < 0.05, |log_2_ FC| > 0.25). **(G)** Volcano plot of ABCA1^+^ vs. ABCA1^−^ monocyte DEGs, with pathways highlighted (ABCA1 gene removed from the figure for better visualization). **(H, I)** Violin plots of AUC-based signature scores in ABCA1^−^ vs. ABCA1^+^ macrophages from liver tissue(left) and independent tumor dataset (right): **(H)** TGF-β signaling; **(I)** M2 polarization. White dots = medians. P values calculated using unpaired two-tailed Wilcoxon tests. **(J, K)** Correlation of the 23-gene mono3 signature **(J)** and ABCA1 gene expression **(K)** with TGF-β signaling (left) and M2 polarization (right) in the TCGA-LIHC cohort. P values based on Pearson correlation.

To validate the CD14^+^ mono3 signature in bulk peripheral blood, we re-analyzed PBMC RNA-seq from 17 HCC patients and 17 healthy donors (13). Twenty-three signature genes were significantly up-regulated in patients (FDR < 0.05, log_2_FC > 0.25; **Figure 6A**; **Supplementary Table S4A**), supporting their potential as blood-based diagnostic makers. Receiver-operating characteristic (ROC) analysis demonstrated that the 23-gene signature robustly discriminated HCC patients from healthy donors (AUC = 0.83; **Figure 6B**). Gene-level ROC analysis ranked PLA2G4A, ABCA1, IFNGR2 and RNF24 among the top discriminative markers (**Supplementary Figure 5A**). These results were further validated in an independent bulk PBMC dataset of 10 HCC patients and 6 healthy donors (GSE58208), which achieved with a high diagnostic accuracy (AUC = 0.90; **Supplementary Figure 5B**).

Next, we examined the prognostic significance of this 23-genes mono3 signature on the TCGA-LIHC cohort, where elevated expression was associated with poorer overall survival (log-rank *P* = 0.04; **Figure 6C**). This analysis also confirmed the independent prognostic value of RNF24 (P = 0.00064) and PLA2G4A (P = 0.0043; **Supplementary Figure 5C**). Additionally, a multivariable Cox regression analysis showed a trend toward association with poorer overall survival (HR = 1.695, 95% CI: 0.842–3.414, p = 0.139), with several individual genes such as RNF24 (HR = 1.887, CI: 1.378–2.583, P = 7.55 × 10^−5^) and CEP170 (HR = 1.681, CI: 1.147–2.465, P = 7.55e × 10^−3^) being independently associated with prognosis (**Figure 6D**).

Among the validated genes, ABCA1, a cholesterol-efflux transporter previously linked to immunosuppressive TAMs (34), showed the greatest fold-change in HCC patients versus healthy controls. Single-cell PBMC analysis confirmed that the frequency of ABCA1^+^ monocytes was highest pre-therapy, declined significantly post-therapy, and approached healthy donor levels (**Figure 6E**, **Supplementary Figure S5D**). Differential-expression analysis revealed 727 DEGs between ABCA1^+^ and ABCA1^−^ monocytes pre-therapy, whereas this difference largely disappeared post-therapy and in healthy controls (**Figure 6F**; **Supplementary Table S4B**). Remarkably, 84 of the 95 genes in the CD14^+^ mono3 signature were among those elevated in the ABCA1^+^ subset. Enrichment analysis indicated that pre-therapy ABCA1^+^ monocytes were characterized by elevated TGF-β signaling and ubiquitin-mediated proteolysis, along with diminished inflammatory signatures, consistent with transcriptional features observed during tumor-associated immune suppression (**Figure 6G**, **Supplementary Figures S5E, F**; **Supplementary Table S4C**).

Within liver tissue, ABCA1^+^ macrophages similarly displayed increased TGF-β signaling, a pattern replicated in an independent scRNA-seq dataset of 12 HCC tumors (3) (**Figure 6H**, **Supplementary Figures S5G, H**). These ABCA1^+^ tumor macrophages also showed significantly higher M2-polarization scores than their ABCA1^−^ counterparts across both dataset (**Figure 6I**). In contrast, no such polarization difference was observed between ABCA1^+^ and ABCA1^-^ monocytes in peripheral blood (**Supplementary Figure S5I**). In the TCGA LIHC cohort, we further investigated whether the CD14^+^ mono3 signature and ABCA1 expression were associated with immunosuppressive signaling pathways. Both the mono3 signature and ABCA1 expression showed significant positive correlations with TGF-β signaling (R = 0.64, P < 2.2 × 10^−16^ and R = 0.23, P = 5.5 × 10^−6^, respectively) and M2 polarization (R = 0.42, P < 2.2 × 10^−16^ and R = 0.11, P = 0.04, respectively) (**Figures 5J, K**). These results extend the immunosuppressive relevance of the mono3 signature and implicate ABCA1 in supporting a tumor-associated TGF-β–dominant, M2-like macrophage phenotype in the tumor microenvironment.

Overall, these data validate the 23-gene CD14^+^ mono3 signature at the bulk level, demonstrate its ability to predict overall survival in TCGA-LIHC, and highlight ABCA1,together with its associated TGF-β signaling activity, as a potential marker of tumor-driven immunosuppression in both peripheral blood and the hepatic immune niche.

## Discussion

Although HCC is known to provoke systemic immunosuppression, studies examining whether curative therapy can reverse these body-wide immune defects are limited (7). We addressed this gap by integrating single-cell transcriptomes of peripheral blood immune cells collected before and after curative therapy with public single-cell dataset from HCC liver tissue. Before tumor removal, circulating leukocytes displayed a transcriptionally quiescent program dominated by TGF-β signaling and ubiquitin-mediated proteolysis, indicating tumor-associated systemic suppression. Curative therapy attenuated these suppressive pathways and partially restored interferon activity, cytotoxic gene expression, and cell–cell communication, although none of these features fully returned to healthy-donor values 3 months after therapy. This immunosuppressive signature was also observed in tumor tissue, suggesting that similar transcriptional patterns occur both locally within the liver and systemically in circulation.

Two pathways were consistently associated with the systemic immunosuppressive profile observed in peripheral immune cells: ubiquitin-mediated proteolysis, elevated across all lineages, and monocyte-focused TGF-β signaling. Both pathways were markedly reduced after tumor removal, yet remained higher than in healthy donors, indicating that HCC projects these suppressive influences beyond the tumor microenvironment into a wider “macro-environment”. Heightened ubiquitin–proteasome activity is known to degrade interferon-stimulated and cytotoxic effector molecules (35, 36), which may help explain the lower IFN signaling activity and NK/T-cell functionality observed pre-therapy. Although curative therapy attenuates both, levels remain above healthy baselines, raising the possibility that these pathways may influence immune recovery after therapy.

Most tumor immunology studies have focused on tumor-infiltrating leukocytes, achieving rich atlases of local anti-tumor immunity (37). However, the tumor-associated environment does not act independently of the periphery immune environment, for example by expanding immature monocytes that subsequently traffic to the tumor and reinforce suppression (38, 39). Here, we bridge these two compartments by identifying a immunosuppressive signature specifically derived from peripheral CD14^+^ mono3 subset, a population enriched before therapy. Notably, this gene signature was also present in an intratumoral macrophage subset, marked by elevated anti–inflammatory genes such as MARCO and CD5L (**Figure 5D**) and exhibiting an M2‐polarized, IL-10–driven profile. Although lineage tracing between public tissue data and our PBMC cohort is not feasible, the convergence of these signatures, suggesting that myeloid cells in blood and liver share immunosuppressive transcriptional features. Notably, the CD14^+^ mono3 signature is accompanied by increased TGF-β signaling in blood and IL-10 signaling in liver tissue, both of which contribute to tumor progression and underscore context-specific immunosuppression (40). Patients exhibiting higher peripheral CD14^+^ mono3-derived signature scores may represent a biologically distinct group, although therapeutic implications require further investigation.

Twenty-three genes from the CD14^+^ mono3 signature were validated in bulk PBMCs, and high expression of this panel predicted poorer overall survival in TCGA-LIHC. RNF24, an E3 ubiquitin ligase previously associated with HCC prognosis, exemplifies the tumor-driven ubiquitination features identified in our study (41). PLA2G4A, which favors M2 polarization, reinforces the link to immunosuppressive reprogramming (42). ABCA1, a cholesterol-efflux transporter critical for the TAMs differentiation (34, 43), emerged as the most robust marker within our signature. In blood, ABCA1^+^ monocytes displayed elevated TGF-β and ubiquitin‐pathway signatures pre-therapy without heightened M2 polarization, whereas ABCA1^+^ macrophages in liver exhibited concurrent increases in TGF-β signaling and M2 polarization. These findings underscore ABCA1 as a potential marker of tumor-associated immunosuppressive states, affecting both systemic circulation and hepatic niche. Supporting this, *in vitro* studies have shown that tumor culture supernatants reduce intracellular cholesterol in macrophages via ABCA1-mediated efflux (34) and cholesterol depletion enhances TGF-β signaling (44), raising the possibility that dysregulated cholesterol homeostasis may contribute to these transcriptional patterns. In HCC specifically, ABCA1^+^ macrophages have been linked to immunosuppression and a poor prognosis (34). Additionally, TGF-β1 has been reported to transcriptionally upregulate ABCA1 via Liver X Receptor alpha signaling in macrophages, supporting a mechanistically plausible connection (45). While our study does not establish a causal role for ABCA1, its consistent association with suppressive transcriptional programs across compartments highlights its potential as a clinically relevant marker of tumor-associated immune modulation.

Our study has several limitations. First, the discovery group was small (n=6) and included patients treated by either ablation (n=5) or surgical resection (n=1) interventions that elicit different inflammatory and immune-recovery pathways. In addition, the underlying etiology of liver disease in our patient cohort was heterogeneous, which may influence immune cell phenotypes, independent of tumor presence. Both groups exhibited comparable immune restoration, indicating that tumor removal, rather than the specific therapeutic approach, may underlie the observed changes. We mitigated the limited sample size through paired pre-/post-sampling, comparison with age-matched healthy donors, and validation in independent bulk-PBMC and tissue datasets; nonetheless, larger cohorts and etiology-matched cohorts are necessary to validate and generalize these findings. Second, our post‐therapy analysis captures only a single time point, which is limited to detect kinetic changes in immune cells profiles. Third, the current study is transcriptome-centered. Incorporation of proteomics, lipidomics, and functional validation will be necessary to confirm causality and uncover post-translational regulation. Finally, the context-dependent role of ABCA1 requires caution in interpretation: whether ABCA1 actively drives immunosuppression or passively marks this state remains to be determined. Addressing these issues in larger, longitudinal, multi-omics cohorts will strengthen the clinical applicability of the signatures described here.

In conclusion, early-stage HCC initiates a systemic immunosuppressive transcriptional program characterized by TGF-β signaling and an expanded suppressive CD14^+^ monocyte subset in peripheral blood, with only partially reversed three months after curative therapy. A distilled, ABCA1−anchored 23−gene signature, detectable in both blood and tumor tissue, reflects these immunosuppressive transcriptional states and may hold potential for future non−invasive diagnostic and prognostic applications pending further validation.

## Data Availability

The raw and processed data from this study have been deposited in the ArrayExpress repository under accession number E-MTAB-16408 and are accessible at https://www.ebi.ac.uk/biostudies/arrayexpress/studies/E-MTAB-16408.
