# Supplementary material for: A monocyte-derived blood transcriptomic signature reveals systemic immunosuppression in HCC and partial reversal following curative therapy
Source: Front Immunol. 2026 Jan 21;16:1717978. doi: 10.3389/fimmu.2025.1717978 (PMC12867797; doi:10.3389/fimmu.2025.1717978)
Supplement: Supplementary file 1 [file DataSheet1.pdf]

(A)

Patient ID	Birth year	Sex	liver disease	Firbrosis/ cirrhosis	liver function	Procedure	Tumor size&number	Pre-therapy sample	Post-therapy sample	Recurrence
HCC1	1953	M	alcoholic	cirrhosis	Child-Pugh A	ablation	33x28mm	14.04.2022	27.05.2022	no
HCC2	1942	M	alcoholic	cirrhosis	Child-Pugh A	ablation	23mm	10.02.2022	01.04.2022	no
HCC3	1957	W	HCV	cirrhosis	Child-Pugh C	ablation	27x30 mm	02.12.2021	11.01.2022	no
HCC4	1960	M	HCV	fibrosis	not applicable	ablation	9mm	18.02.2022	21.09.2022	no
HCC5	1949	M	MASLD	steatosis	not applicable	resection	3 tumors: 29mm, 18mm, 17mm	17.03.2022	24.05.2022	no
HCC6	1965	M	alcoholic	cirrhosis	Child-Pugh A	ablation	22.01: 32mm, 22.03: 12mm	11.01.2022	22.04.2022	no

(B)

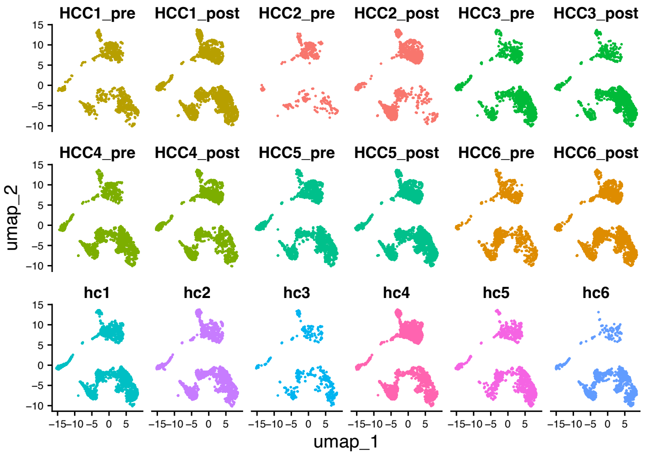

(C)

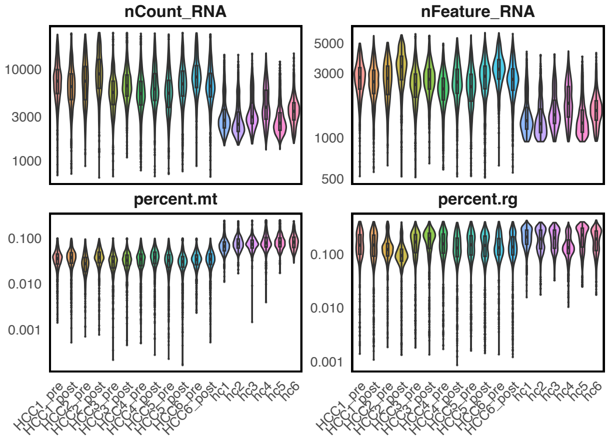

(D)

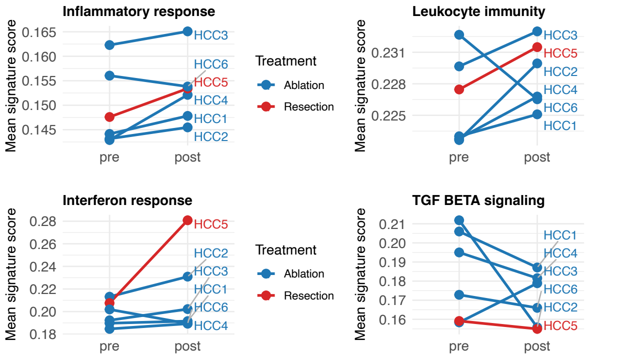

(E)

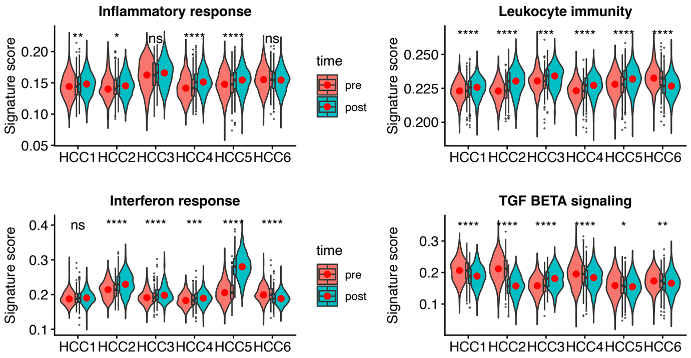

(F)

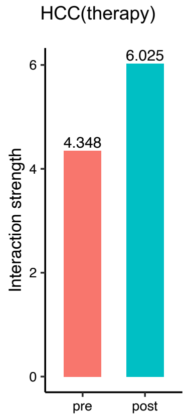

(G)

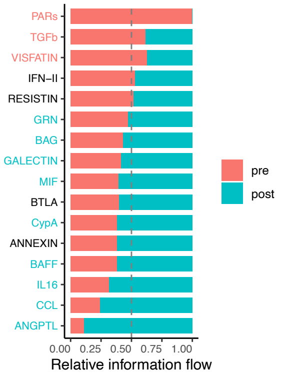

SUPPLEMENT FIGURE S1

Supplementary Figure S1. Clinical information and comparative profiling of peripheral immune cells. (A) Clinical characteristics of six HCC patients. (B) UMAP visualization showing balanced cellular representation across all individuals and conditions. Cells are colored by patient ID for both pre- and post-therapy PBMC samples, as well as healthy donors. (C) Violin plots displaying quality control metrics across all samples, including total RNA counts (nCount_RNA), number of detected features (nFeature_RNA), mitochondrial gene percentage (percent.mt), and ribosomal gene percentage (percent.rp). (D) AUC scores for four key immune signatures (inflammatory response, leukocyte immunity, interferon response, and TGF-β signaling) across patients, shown pre- and post-therapy. Lines indicate per-patient changes; colors indicate treatment type. (E) Violin plots showing AUC scores for each immune signature; P values from unpaired two-tailed Wilcoxon tests. (F) Aggregate CellChat interaction strength (sum of incoming + outgoing signals) pre vs. post; numbers indicate total strength. (G) Relative information flow of top signaling pathways in pre and post; significant pathways labeled (red = pre, blue = post).

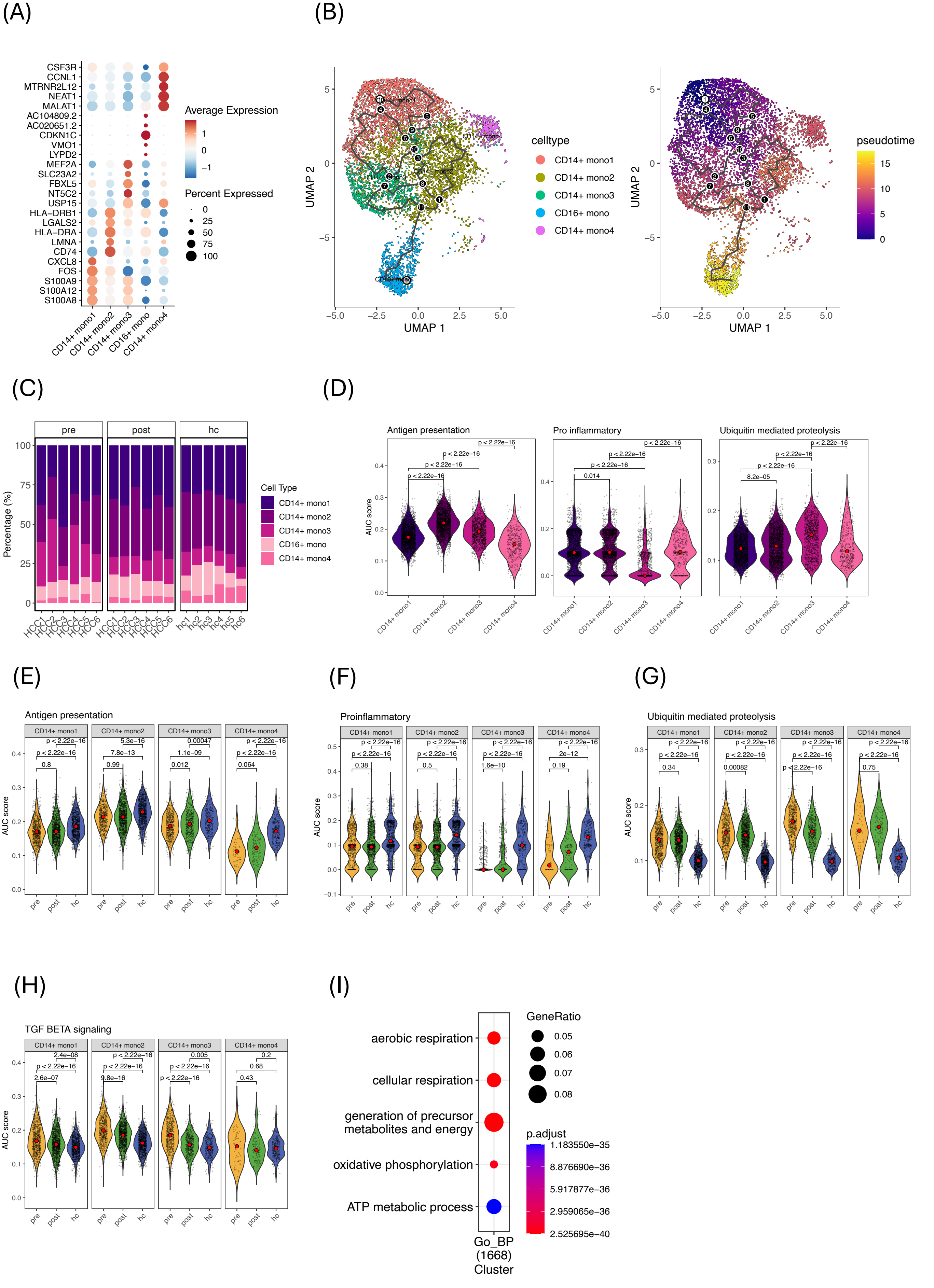

SUPPLEMENT FIGURE S2

Supplementary Figure S2. Detailed analyses of circulating monocyte subsets in HCC patients. (A) Dot plot of marker genes for five monocyte subsets; size = % expressing cells, color = average expression. (B) Trajectory analysis colored by subset (left) and pseudotime (right); mono1 root diverges into mono2 and mono3 then converges on $CD16^+$ mono. (C) Per-patient monocyte subset composition in pre, post, and hc samples. (D) Violin plots of AUC-based signature scores for antigen presentation, pro-inflammatory, and ubiquitin-mediated proteolysis across $CD14^+$ mono1–mono4; red dots = medians. (E–H) Violin plots of AUC-based signature scores across $CD14^+$ mono1–mono4 under pre, post, and hc: (E) Antigen presentation, (F) Pro-inflammatory, (G) Ubiquitin-mediated proteolysis, (H) TGF-β signaling. (I) GO terms enriched in $CD14^+$ mono4 genes. All P values calculated using unpaired two-tailed Wilcoxon tests.

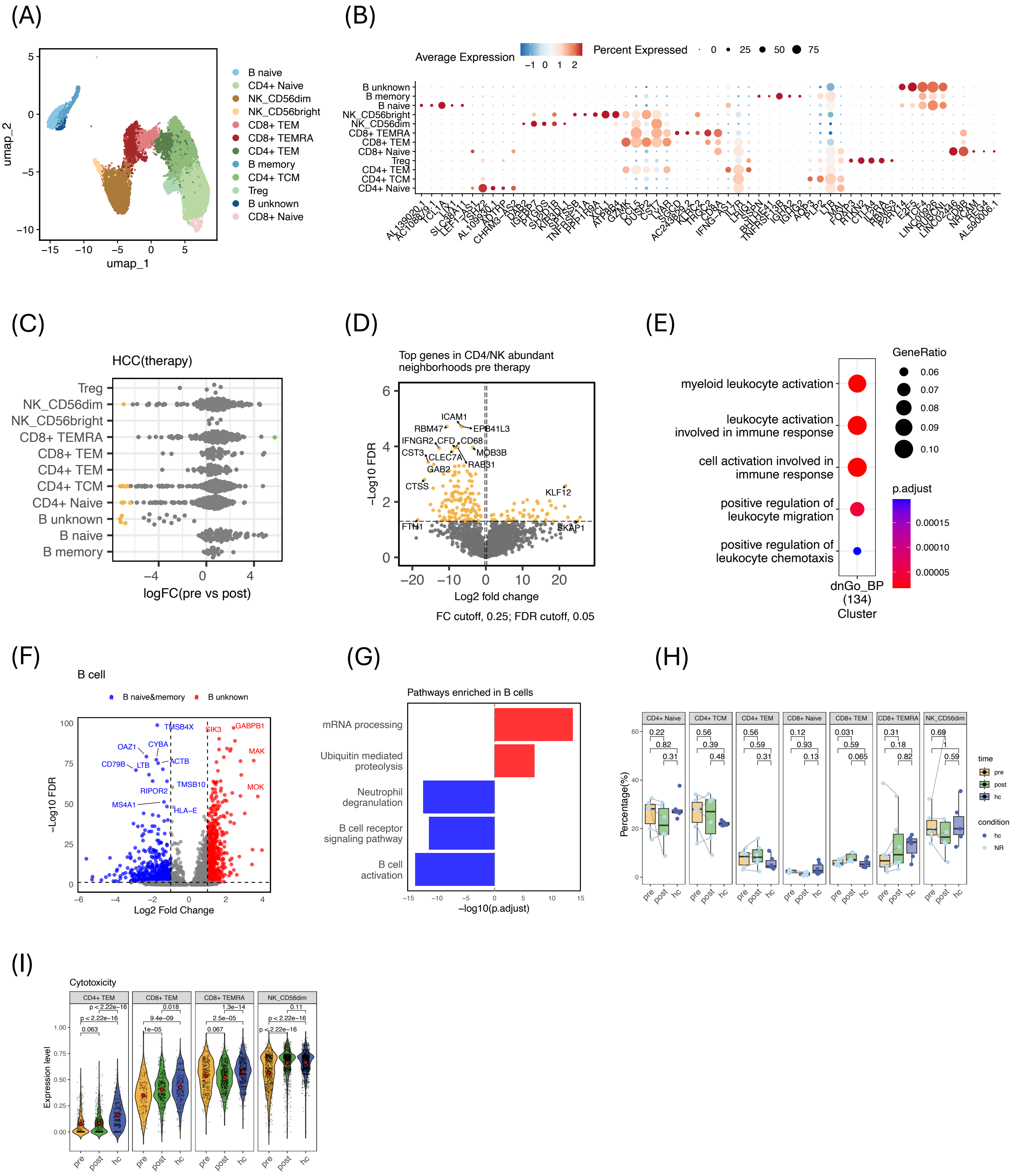

SUPPLEMENT FIGURE S3

Supplementary Figure S3. Lymphocyte heterogeneity and tumor-associated suppression in peripheral blood. (A) UMAP of lymphocytes resolving four $CD4^+$ T, three $CD8^+$ T, two NK, and three B cell subsets. (B) Dot plot of marker genes per subset; size = % expressing cells, color = average expression. (C) Differential abundance analysis with MiloR; x = $\log_2$ FC (pre vs. post), y = $-\log_{10}$ FDR. (D) Top genes driving pre-therapy enrichment in $CD4^+$ naïve/TCM/NK neighborhoods (FDR < 0.05, $|\log_2 FC| > 0.25$). (E) GO enrichment for 134 genes depleted in (D). (F) Volcano plot comparing "B unknown" subset vs. other B cells. (G) Pathway enrichment for genes down (blue) or up (red) regulated in "B unknown." (H) Frequencies of effector/activated lymphocyte subsets pre vs. post. P values were calculated using paired two-tailed Wilcoxon test (pre vs. post) or unpaired two-tailed Wilcoxon tests (hc vs. pre/post), as indicated. (I) Violin plots of AUC-based cytotoxicity signature scores in effector $CD4^+$, $CD8^+$, and NK cells; medians indicated by horizontal bars. P values calculated using unpaired two-tailed Wilcoxon tests.

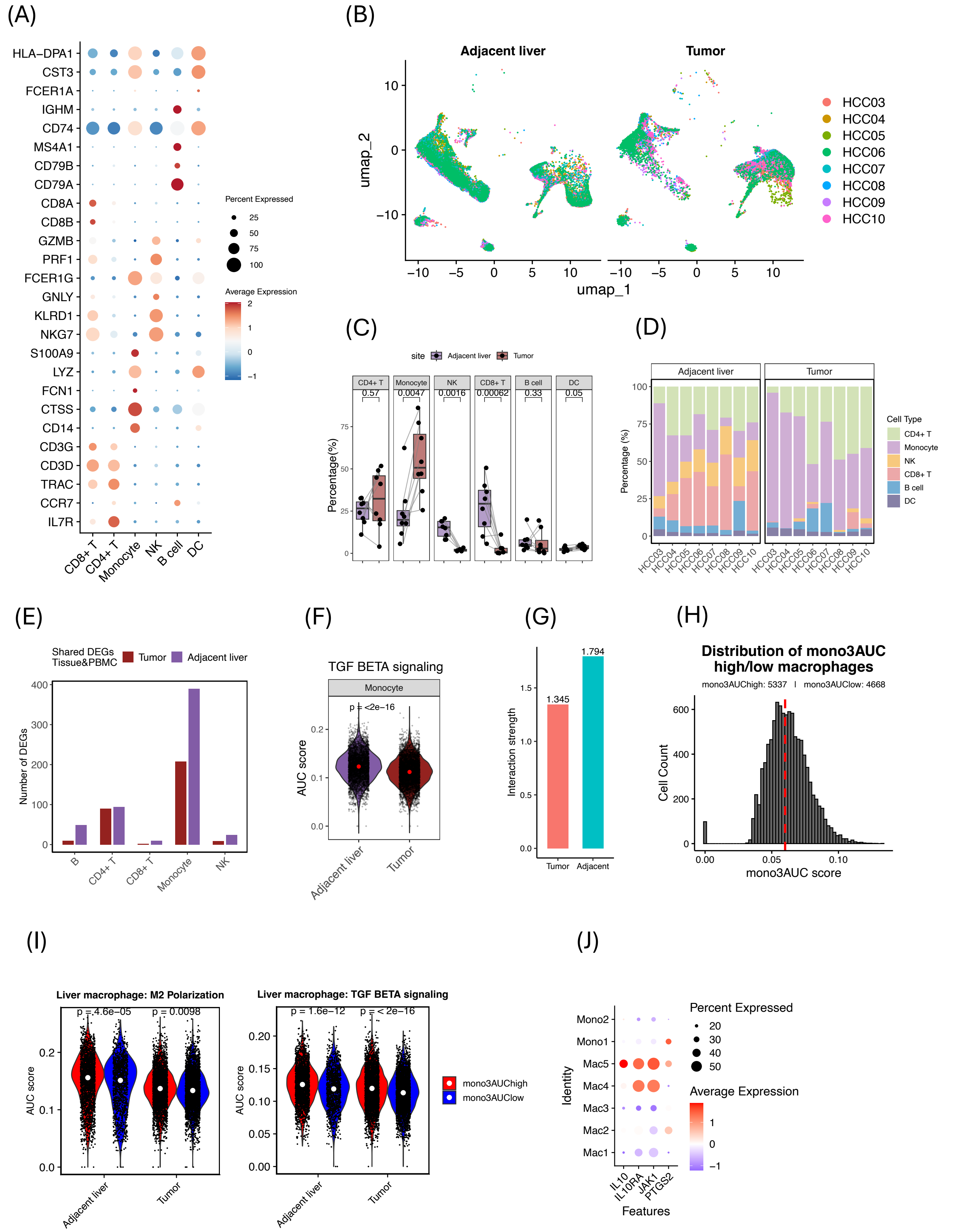

SUPPLEMENT FIGURE S4

Supplementary Figure S4. Comparative profiling of immune cells from tumor and adjacent liver tissue. (A) Dot plot of marker genes for tissue immune lineages; size = % expressing cells, color = average expression. (B) UMAP colored by donor, confirming balanced representation. (C) Box plots of lineage frequencies (adjacent liver vs. tumor); paired lines connect samples. (D) Stacked bars of per-patient immune composition. (E) Overlap between site-specific DEGs and pre vs. post PBMC DEGs. (F) Violin plot of AUC-based TGF-β signature scores in monocytes (adjacent vs. tumor). (G) Aggregate CellChat interaction strength tumor vs. adjacent liver. (H) Histogram of mono3AUC score in primary tumor macrophages; red dashed line = mono3AUChigh cutoff (n = 5,337), below = mono3AUClow (n = 4,668). (I) Violin plots of AUC-based M2 polarization and TGF-β signaling scores. (J) Expression of IL10, IL10RA, JAK2, PTGS2 across macrophage/monocyte subsets. All P values calculated using unpaired two-tailed Wilcoxon tests.

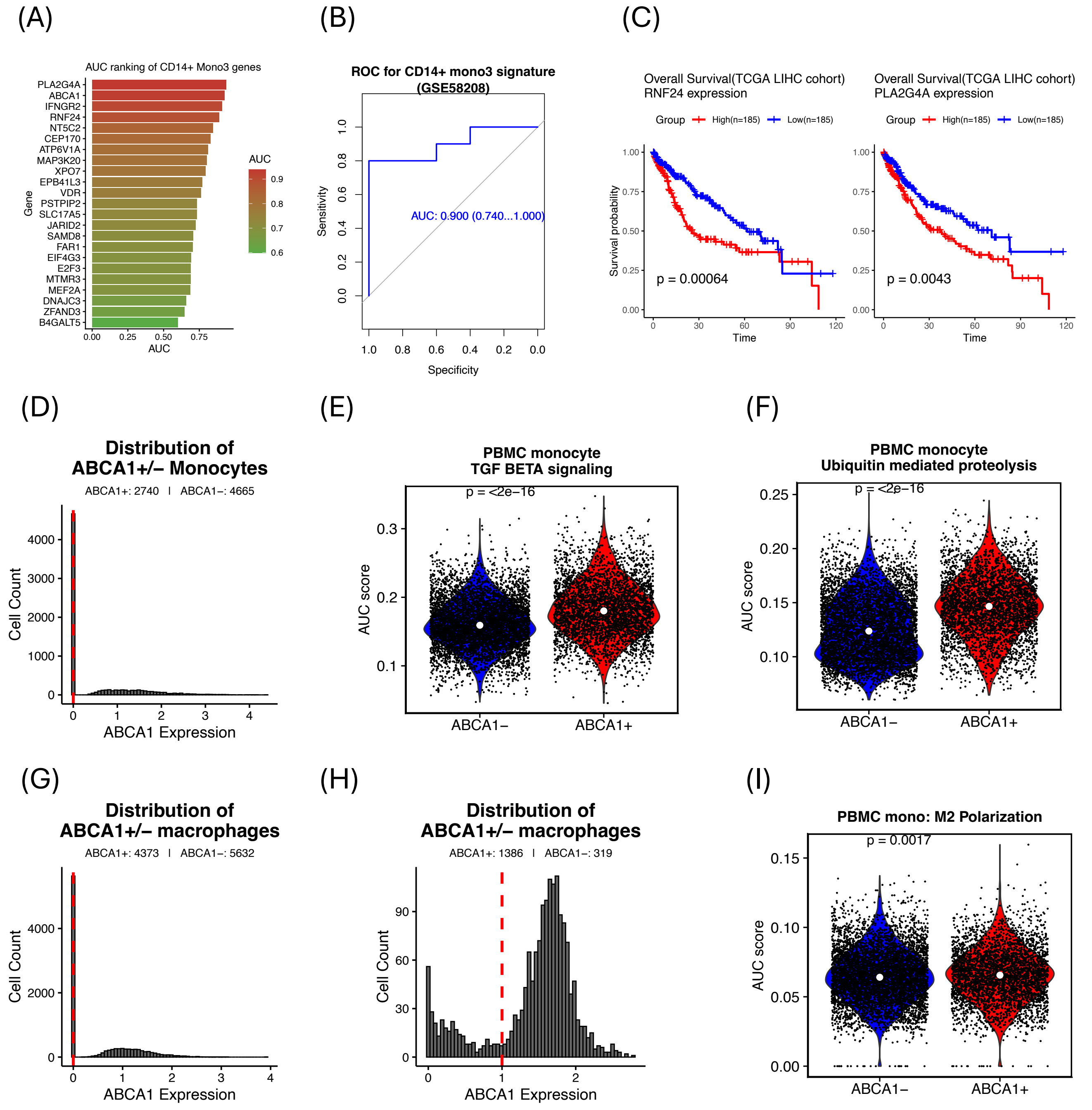

SUPPLEMENT FIGURE S5

Supplementary Figure S5. Definition and functional characterization of $ABCA1^+$ and $ABCA1^-$ myeloid cells. (A) ROC-based AUC ranking of individual genes within the 23-gene mono3 signature based on their diagnostic performance. (B) ROC curve showing diagnostic accuracy of the 23-gene mono3 signature in an independent PBMC dataset (GSE58208). (C) Kaplan–Meier survival curves (TCGA-LIHC) for RNF24 and PLA2G4A expression (high vs. low; log-rank P values shown). (D) Histogram of ABCA1 expression in PBMC monocytes (pre + post + HC); red dashed line = $ABCA1^+$ cutoff (n = 2,740), below = $ABCA1^-$ (n = 4,665). (E–F) Violin plots of AUC-based TGF-β signaling (E) and ubiquitin-mediated proteolysis (F) scores in $ABCA1^-$ vs. $ABCA1^+$ PBMC monocytes; white dots = medians. (G) Histogram of ABCA1 in primary tumor macrophages; red dashed line = $ABCA1^+$ cutoff (n = 4,373), below = $ABCA1^-$ (n = 5,632). (H) Histogram of ABCA1 in independent tumor cohort (n = 12); red dashed line = $ABCA1^+$ cutoff (n = 1,386), below = $ABCA1^-$ (n = 319). (I) Violin plot of AUC-based M2 polarization scores in $ABCA1^-$ vs. $ABCA1^+$ PBMC monocytes; white dots = medians. All P values calculated using unpaired two-tailed Wilcoxon tests
